# Supplementary material for: Near-room-temperature martensitic actuation profited from one-dimensional hybrid perovskite structure
Source: Nat Commun. 2022 Nov 3;13:6599. doi: 10.1038/s41467-022-34356-9 (PMC9633747; doi:10.1038/s41467-022-34356-9)
Supplement: Supplementary file 1 — Supplementary Information [file 41467_2022_34356_MOESM1_ESM.pdf]

Supplementary Information

Near-room-temperature martensitic actuation profited from one-dimensional hybrid perovskite structure

Liang et al.

Contents

Supplementary Note 1 | Spontaneous strain calculation.

Supplementary Fig. 1 | Experimental and simulated PXRD patterns for powder sample of **1** in the HTP.

Supplementary Fig. 2 | TGA measurement of **1**. The decomposition begins at about 500 K.

Supplementary Fig. 3 | Single crystal orientation.

Supplementary Fig. 4 | Dielectric constant curves of **1** at different frequencies.

Supplementary Fig. 5 | Hirshfeld surfaces for **1** of $[PbI_3]_n$.

Supplementary Fig. 6 | Order-disorder change of cations during the phase transitions. The NMEA cation in thermal ellipsoid mode with 50% probability.

Supplementary Fig. 7 | Phase boundary movement. The movement was observed in the (100) when the crystal undergoes the transition from the HTP to ITP.

Supplementary Fig. 8 | Temperature-dependent resistivity of **1**.

Supplementary Table 1 | Crystallographic data and structure refinement details for **1** at different phases.

Supplementary Table 2 | I-Pb bond lengths of **1** in the LTP, ITP, and HTP.

Supplementary Table 3 | Converted cell parameters for spontaneous strain calculation.

Supplementary Note 1 | Spontaneous strain calculation.

General equations for the components of the spontaneous strain tensor have the following expression:

$$x_{ij}=\begin{bmatrix} \frac{a\sin\gamma}{a_0\sin\gamma_0}-1 & \frac{1}{2}\left[\frac{a\cos\gamma}{a_0\sin\gamma_0}-\frac{b\cos\gamma_0}{b_0\cos\gamma_0}\right] & \frac{1}{2}\left[\frac{a\sin\gamma\cos{\beta_0}^*}{a_0\sin\gamma_0\sin{\beta_0}^*}-\frac{c\sin\alpha\cos\beta^*}{c_0\sin\alpha_0\sin{\beta_0}^*}\right] \\ \frac{1}{2}\left[\frac{a\cos\gamma}{a_0\sin\gamma_0}-\frac{b\cos\gamma_0}{b_0\cos\gamma_0}\right] & \frac{b}{b_0}-1 & \frac{1}{2}\left[\frac{c\cos\alpha}{c_0\sin\alpha_0\sin{\beta_0}^*}-\frac{b\cos\alpha_0}{b_0\sin\alpha_0\sin{\beta_0}^*}+\frac{\cos{\beta_0}^*}{\sin{\beta_0}^*\sin\gamma_0}\left(\frac{a\cos\gamma}{a_0}-\frac{b\cos\gamma_0}{b_0}\right)\right] \\ \frac{1}{2}\left[\frac{a\sin\gamma\cos{\beta_0}^*}{a_0\sin\gamma_0\sin{\beta_0}^*}-\frac{c\sin\alpha\cos\beta^*}{c_0\sin\alpha_0\sin{\beta_0}^*}\right] & \frac{1}{2}\left[\frac{c\cos\alpha}{c_0\sin\alpha_0\sin{\beta_0}^*}-\frac{b\cos\alpha_0}{b_0\sin\alpha_0\sin{\beta_0}^*}+\frac{\cos{\beta_0}^*}{\sin{\beta_0}^*\sin\gamma_0}\left(\frac{a\cos\gamma}{a_0}-\frac{b\cos\gamma_0}{b_0}\right)\right] & \frac{c\sin\alpha\sin\beta^*}{c_0\sin\alpha_0\sin{\beta_0}^*}-1 \end{bmatrix}$$

In the matrix, a, b, c, α, β^*, and γ represent the cell parameters at low-symmetry, and a_0, b_0, c_0, α_0, β_0, and γ_0 are the cell parameters transformed from the high symmetry form based on low-symmetry form. β^* and β_0^* are the reciprocal lattice angles.

In the ITP, spontaneous strain tensors X_S for the six possible orientation states are given as:

$$X_s(S_1)=\begin{bmatrix} -w & 0 & x_{13} \\ 0 & w & 0 \\ x_{13} & 0 & 0 \end{bmatrix},\ X_s(S_2)=\begin{bmatrix} -w & 0 & -x_{13} \\ 0 & w & 0 \\ -x_{13} & 0 & 0 \end{bmatrix}$$

$$X_s(S_3)=\begin{bmatrix} \frac{1}{2}w & \frac{\sqrt{3}}{2}w & -\frac{1}{2}x_{13} \\ \frac{\sqrt{3}}{2}w & -\frac{1}{2}w & \frac{\sqrt{3}}{2}x_{13} \\ -\frac{1}{2}x_{13} & \frac{\sqrt{3}}{2}x_{13} & 0 \end{bmatrix},\ X_s(S_4)=\begin{bmatrix} \frac{1}{2}w & -\frac{\sqrt{3}}{2}w & \frac{1}{2}x_{13} \\ -\frac{\sqrt{3}}{2}w & -\frac{1}{2}w & \frac{\sqrt{3}}{2}x_{13} \\ \frac{1}{2}x_{13} & \frac{\sqrt{3}}{2}x_{13} & 0 \end{bmatrix}$$

$$X_s(S_5)=\begin{bmatrix} \frac{1}{2}w & -\frac{\sqrt{3}}{2}w & -\frac{1}{2}x_{13} \\ -\frac{\sqrt{3}}{2}w & -\frac{1}{2}w & -\frac{\sqrt{3}}{2}x_{13} \\ -\frac{1}{2}x_{13} & -\frac{\sqrt{3}}{2}x_{13} & 0 \end{bmatrix}\ \text{and}\ X_s(S_6)=\begin{bmatrix} \frac{1}{2}w & \frac{\sqrt{3}}{2}w & \frac{1}{2}x_{13} \\ \frac{\sqrt{3}}{2}w & -\frac{1}{2}w & -\frac{\sqrt{3}}{2}x_{13} \\ \frac{1}{2}x_{13} & -\frac{\sqrt{3}}{2}x_{13} & 0 \end{bmatrix}$$

In the LTP, spontaneous strain tensors X_S for the two possible orientation states are given as:

$$X_s(S_1)=\begin{bmatrix} 0 & x_{12} & 0 \\ x_{12} & 0 & x_{23} \\ 0 & x_{23} & 0 \end{bmatrix}\ \text{and}\ X_s(S_2)=\begin{bmatrix} 0 & -x_{12} & 0 \\ -x_{12} & 0 & -x_{23} \\ 0 & -x_{23} & 0 \end{bmatrix}$$

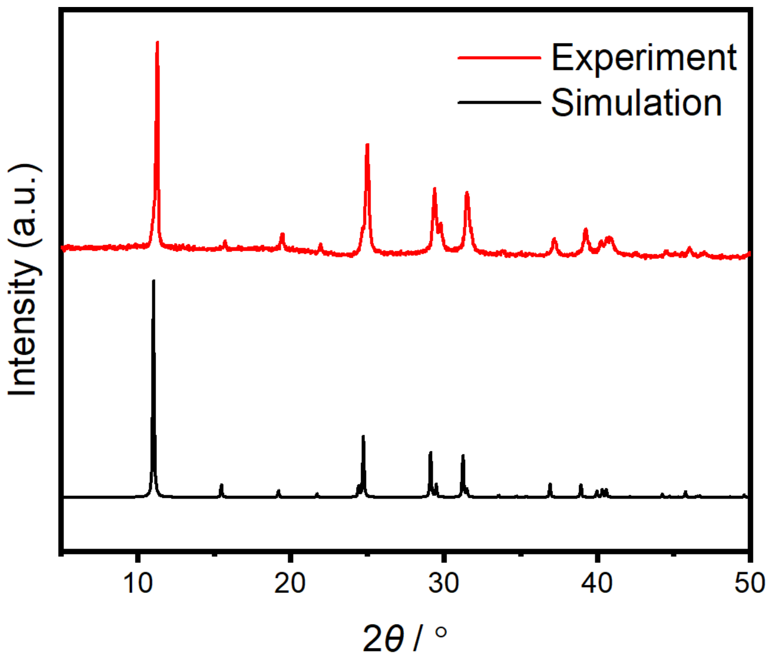

Supplementary Fig. 1 | Experimental and simulated PXRD patterns for powder sample of 1 in the HTP. Source data are provided as a Source Data file.

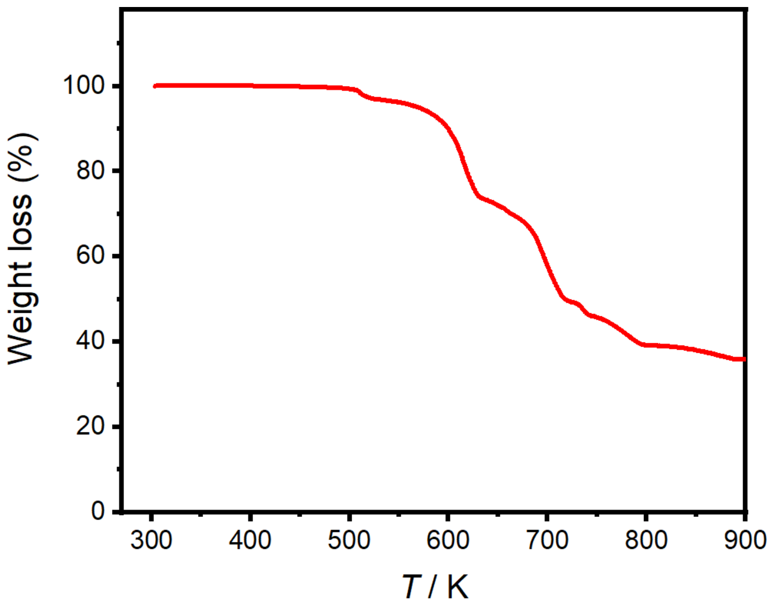

Supplementary Fig. 2 | TGA measurement of 1. The decomposition begins at about 500 K. Source data are provided as a Source Data file.

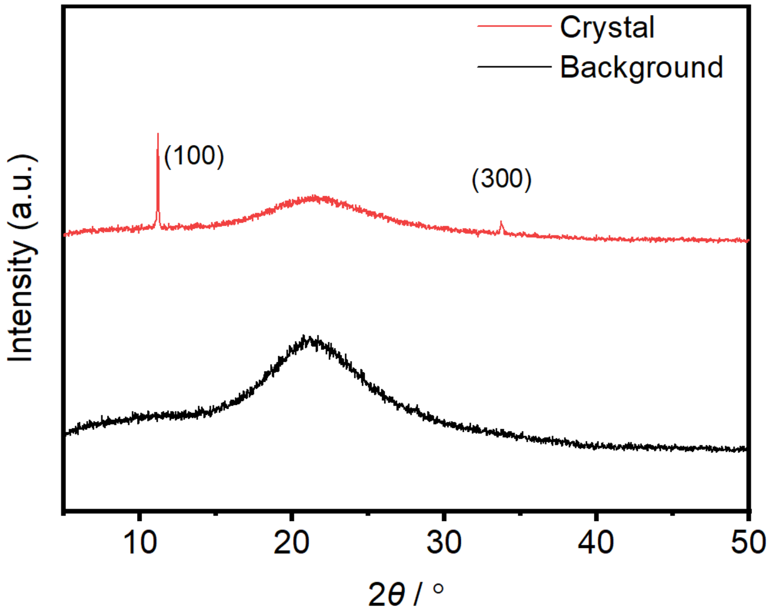

Supplementary Fig. 3 | Single crystal orientation. Source data are provided as a Source Data file.

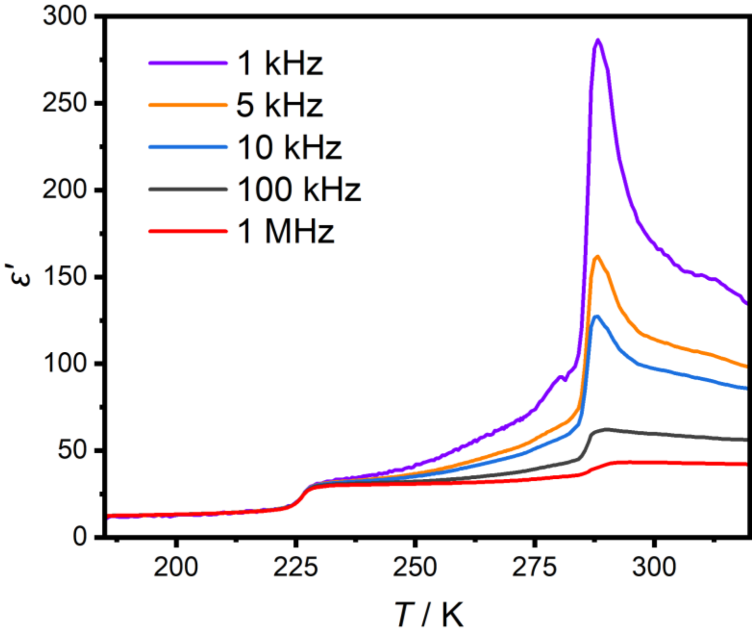

Supplementary Fig. 4 | Dielectric constant curves of 1 at different frequencies. Source data are provided as a Source Data file.

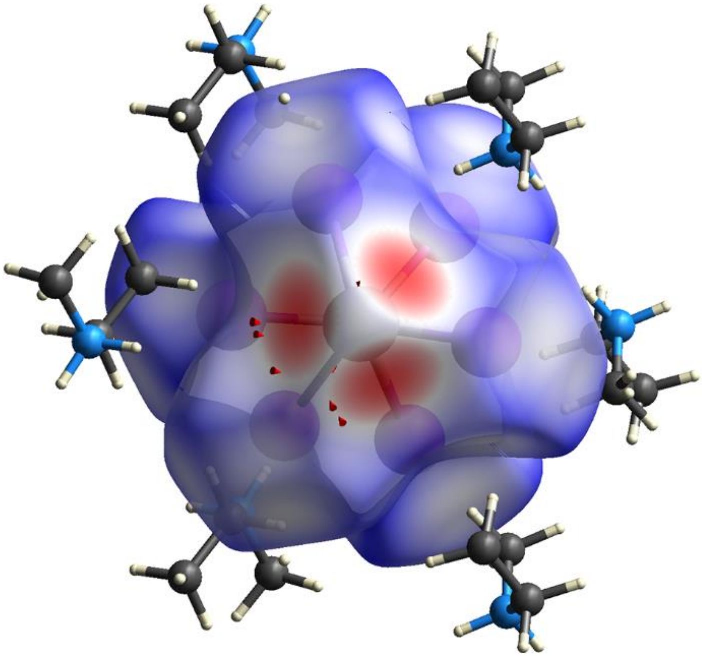

Supplementary Fig. 5 | Hirshfeld surfaces for 1 of $[PbI_3]_n$.

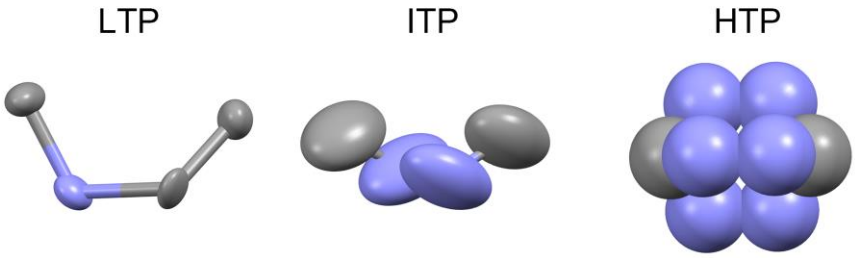

Supplementary Fig. 6 | Order-disorder change of cations during the phase transitions. The NMEA cation in thermal ellipsoid mode with 50% probability.

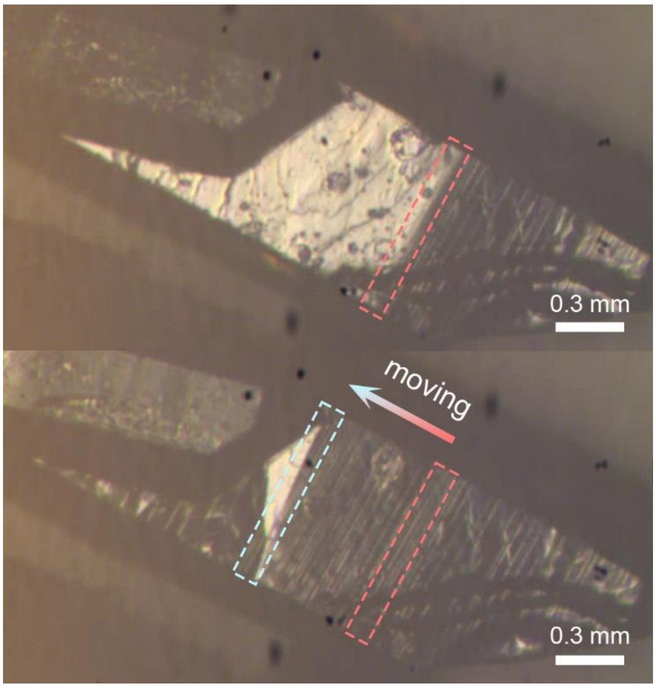

Supplementary Fig. 7 | Phase boundary movement. The movement was observed in the (100) when the crystal undergoes the transition from the HTP to ITP.

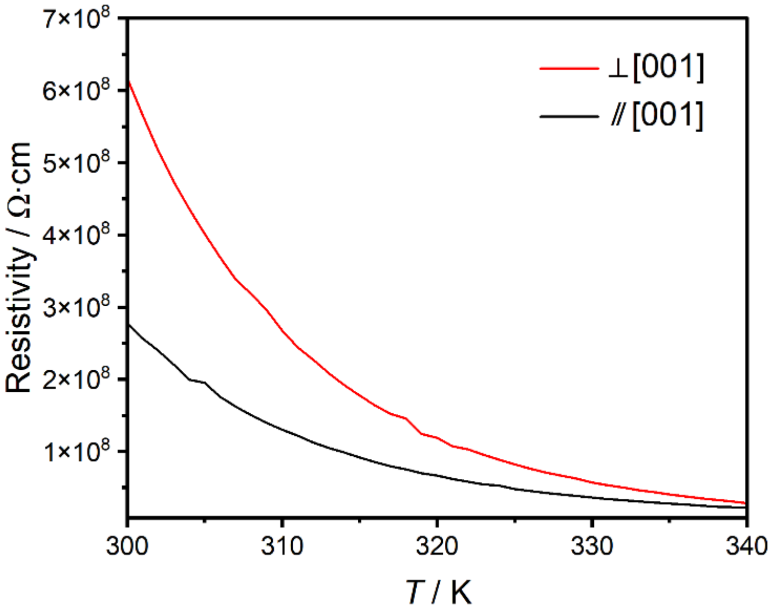

Supplementary Fig. 8 | Temperature-dependent resistivity of 1. Source data are provided as a Source Data file.

Supplementary Table 1 | Crystallographic data and structure refinement details for 1 at different phases.

	**LTP-173K**	**ITP-253K**	**HTP-333K**
Formula	$C_6H_{20}N_2I_6Pb_2$	$C_3H_{10}NI_3Pb$	$C_3H_{10}NI_3Pb$
Formula weight	1296.04	648.02	648.02
Crystal system	Triclinic	Monoclinic	Hexagonal
space group	*P*-1	*C*2/*c*	$P6_3/mmc$
a / Å	8.1501(3)	9.8984(9)	9.2432(5)
b / Å	9.0775(2)	15.0923(10)	9.2432(5)
c / Å	15.8322(3)	8.0597(5)	8.1797(4)
α / °	83.8690(10)	90	90
β / °	84.399(2)	97.887(7)	90
γ / °	87.646(2)	90	120
V / $Å^3$	1158.49(5)	1192.65(15)	605.22(7)
Z	2	4	2
D_{calc} / $g\cdot cm^{-3}$	3.715	3.609	3.501
μ / mm^{-1}	22.495	21.851	21.527
Reflns. collected	28600	6472	6429
Obsd. reflns. [I>2σ(I)]	5081	1133	202
R_{int}	0.0493	0.0432	0.0777
R_1^a/wR_2^b [I>2σ(I)]	0.0599,0.1654	0.0516,0.1512	0.0577,0.1694
R_1/wR_2 (all data)	0.0679,0.1678	0.0690,0.1591	0.0931,0.1950
GOF	1.186	1.103	1.035
$\Delta\rho^c$ / $e\cdot Å^{-3}$	3.247/-3.304	2.606/-0.996	0.654/-0.995

[a] $R_1 = \Sigma||F_o| - |F_c||/\Sigma|F_o|$. [b] $wR_2(F^2) = [\Sigma w(F_o^2 - F_c^2)^2/\Sigma wF_o^4]^{1/2}$. [c] Maximum and minimum residual electron density.

Supplementary Table 2 | I-Pb bond lengths of 1 in the LTP, ITP, and HTP.

Bond lengths	Length / Å
**LTP-173K**	
I(5)−Pb(1)	3.4007(10)
I(6)−Pb(1)^{ii}	3.3409(11)
I(3)−Pb(2)	3.3741(10)
I(1)−Pb(2)^{i}	3.3100(11)
I(2)−Pb(2)^{i}	3.2564(11)
I(2)−Pb(1)	3.2355(11)
I(4)−Pb(2)	3.2192(10)
I(4)−Pb(1)	3.2143(11)
I(5)−Pb(2)	3.1664(10)
I(3)−Pb(1)	3.1235(10)
I(1)−Pb(1)	3.1036(10)
I(6)−Pb(2)	3.0992(10)
Symmetry codes: (i) −1+x, +y, +z; (ii) 1+x, +y, +z.	
**ITP-253K**	
I(2)−Pb(1)^{i}	3.2394(12)
I(2)−Pb(1)	3.2227(10)
I(1)−Pb(1)	3.2047(10)
I(1)−Pb(1)^{i}	3.2047(10)
Symmetry codes: (i) 1−x, +y, 3/2−z.	
**HTP-333K**	
I(1)−Pb(1)	3.2249(19)
I(1)−Pb(1)^{i}	3.2249(19)
Symmetry codes: (i) −x, −y, 1/2+z.	

Supplementary Table 3 | Converted cell parameters for spontaneous strain calculation.

	**LTP-ITP Transition**	**ITP-HTP Transition**
a / Å	9.0775(2)	9.8984(9)
b / Å	15.8322(3)	15.0923(10)
c / Å	8.1501(3)	8.0597(5)
α / °	95.601(2)	90
β / °	87.646(2)	97.887(7)
γ / °	96.310(10)	90
a_0 / Å	9.8984(9)	9.2432(5)
b_0 / Å	15.0923(10)	16.0097(9)
c_0 / Å	8.0597(5)	8.1797(4)
α_0 / °	90	90
β_0 / °	97.887(7)	90
γ_0 / °	90	90
