# Supplementary material for: Near-room-temperature martensitic actuation profited from one-dimensional hybrid perovskite structure
Source: Nat Commun. 2022 Nov 3;13:6599. doi: 10.1038/s41467-022-34356-9 (PMC9633747; doi:10.1038/s41467-022-34356-9)
Supplement: Supplementary file 3 — Description of Additional Supplementary Files [file 41467_2022_34356_MOESM3_ESM.pdf]

Description of Additional Supplementary Files

Supplementary Movie 1 | Evolution of ferroelastic domain structures.

Supplementary Movie 2 | Mechanically induced crystal twinning.

Supplementary Movie 3 | Crystal morphology evolution during martensitic phase transitions.

Supplementary Movie 4 | Crystal morphology evolution during martensitic phase transitions.

Supplementary Movie 5 | Phase boundary movement.

Supplementary Movie 6 | Demonstration of the temperature-controlled mechanical switch.
